# Supplementary material for: How do multi-morbidity and polypharmacy affect general practice attendance and referral rates? A retrospective analysis of consultations
Source: PLoS One. 2022 Feb 3;17(2):e0263258. doi: 10.1371/journal.pone.0263258 (PMC8812985; doi:10.1371/journal.pone.0263258)
Supplement: S1 Table — (PDF) [file pone.0263258.s001.pdf]

List of chronic conditions

Affective (mood) disorder

Asthma

Bronchiectasis

Cancer

Cardiomyopathy

Cardiac failure

Chronic kidney disease

Chronic obstructive pulmonary disease

Coronary artery disease

Crohn’s disease/ other inflammatory bowel condition

Dementia

Diabetes mellitus (types 1&2)

Dysrhythmia

Endocrine - other

Epilepsy

Glaucoma

Haemophilia

Hyperlipidaemia

Hypertension

Multiple sclerosis

Osteoarthritis

Osteoporosis

Parkinson’s Disease

Rheumatoid arthritis/ other inflammatory arthritis

Schizophrenia/ other psychosis

Systemic lupus erythematosus/ other inflammatory condition

Thyroid condition
