# Supplementary material for: How do multi-morbidity and polypharmacy affect general practice attendance and referral rates? A retrospective analysis of consultations
Source: PLoS One. 2022 Feb 3;17(2):e0263258. doi: 10.1371/journal.pone.0263258 (PMC8812985; doi:10.1371/journal.pone.0263258)
Supplement: Attachment — Submitted filename: Response_to_Reviewers.docx [file pone.0263258.s002.docx]

19.08.2021

Dear editor and reviewers,

We are very grateful for your time in reading and reviewing this manuscript. We believe that the suggestions received are valuable and we have responded to each of them in detail below. For each response, the corresponding alteration in the manuscript in quoted, where appropriate.

We would like to point out that this was a retrospective analysis of patient records. Each one of the 72 practices affiliated with the Medical School that had a medical student on placement was invited to participate and 68 of them accepted. Of the 6800 records a small percentage were discounted for not having adequate demographic data.

We had full ethical approval for the study. No anonymised data left the practice at any stage and no approval exists for publishing the dataset.

Statistical queries, including confidence intervals and use of Cramer’s V test, have been addressed.

Finally, the research team has a prior publication from this dataset, which has been referenced, and which gives more detail on the destination of the referrals, including hospital specialty.

We hope that the comments have been addressed to the satisfaction of the reviewers.

Best wishes,

Dr Andrew O’Regan, GP and senior lecturer in general practice, on behalf of the research team

Journal Requirements:

When submitting your revision, we need you to address these additional requirements.

1. Please ensure that your manuscript meets PLOS ONE's style requirements, including those for file naming. The PLOS ONE style templates can be found at

https://journals.plos.org/plosone/s/file?id=wjVg/PLOSOne_formatting_sample_main_body.pdf and

[https://journals.plos.org/plosone/s/file?id=ba62/PLOSOne_formatting_sample_title_authors_affiliations.pdf](about:blank)

2.  Please provide additional details regarding participant consent. In the ethics statement in the Methods and online submission information, please ensure that you have specified (a) whether consent was informed and (b) what type you obtained (for instance, written or verbal, and if verbal, how it was documented and witnessed). If your study included minors, state whether you obtained consent from parents or guardians. If the need for consent was waived by the ethics committee, please include this information.

If you are reporting a retrospective study of medical records or archived samples, please ensure that you have discussed whether all data were fully anonymized before you accessed them and/or whether the IRB or ethics committee waived the requirement for informed consent. If patients provided informed written consent to have data from their medical records used in research, please include this information.

Response of the authors: In the subsection ‘Study Population’ of ‘Materials and Methods’, the authors have clarified as follows:

“This study was a retrospective analysis of consultations and was granted full ethical approval by the Irish College of General Practitioners Research Ethics Committee (ICGP, 09/05/2015). As per the ethics application, all data were fully anonymised before leaving the practice and before being accessed for analysis. Individual informed consent was not deemed necessary by the ethics committee.”

3. We note that you have indicated that data from this study are available upon request. PLOS only allows data to be available upon request if there are legal or ethical restrictions on sharing data publicly. For information on unacceptable data access restrictions, please see http://journals.plos.org/plosone/s/data-availability#loc-unacceptable-data-access-restrictions.

In your revised cover letter, please address the following prompts:

a) If there are ethical or legal restrictions on sharing a de-identified data set, please explain them in detail (e.g., data contain potentially identifying or sensitive patient information) and who has imposed them (e.g., an ethics committee). Please also provide contact information for a data access committee, ethics committee, or other institutional body to which data requests may be sent.

Response of the authors: sharing of this dataset is restricted by the Irish College of General Practitioners research ethics committee. Access to the dataset is available upon reasonable request to the Irish College of General Practitioners Research Ethics Committee (research@icgp.ie).

b) If there are no restrictions, please upload the minimal anonymized data set necessary to replicate your study findings as either Supporting Information files or to a stable, public repository and provide us with the relevant URLs, DOIs, or accession numbers. Please see http://www.bmj.com/content/340/bmj.c181.long for guidelines on how to de-identify and prepare clinical data for publication. For a list of acceptable repositories, please see http://journals.plos.org/plosone/s/data-availability#loc-recommended-repositories.

We will update your Data Availability statement on your behalf to reflect the information you provide.

 Response of the authors: same as point a) above.

[Note: HTML markup is below. Please do not edit.]

Reviewers' comments:

Reviewer's Responses to Questions

**Comments to the Author**

1. Is the manuscript technically sound, and do the data support the conclusions?

The manuscript must describe a technically sound piece of scientific research with data that supports the conclusions. Experiments must have been conducted rigorously, with appropriate controls, replication, and sample sizes. The conclusions must be drawn appropriately based on the data presented.

Reviewer #1: Yes

Reviewer #2: Yes

2. Has the statistical analysis been performed appropriately and rigorously?

Reviewer #1: Yes

Reviewer #2: Yes

3. Have the authors made all data underlying the findings in their manuscript fully available?

The [PLOS Data policy](http://www.plosone.org/static/policies.action#sharing) requires authors to make all data underlying the findings described in their manuscript fully available without restriction, with rare exception (please refer to the Data Availability Statement in the manuscript PDF file). The data should be provided as part of the manuscript or its supporting information, or deposited to a public repository. For example, in addition to summary statistics, the data points behind means, medians and variance measures should be available. If there are restrictions on publicly sharing data—e.g. participant privacy or use of data from a third party—those must be specified.

Reviewer #1: No

Reviewer #2: Yes

4. Is the manuscript presented in an intelligible fashion and written in standard English?

PLOS ONE does not copyedit accepted manuscripts, so the language in submitted articles must be clear, correct, and unambiguous. Any typographical or grammatical errors should be corrected at revision, so please note any specific errors here.

Reviewer #1: Yes

Reviewer #2: Yes

5. Review Comments to the Author

Please use the space provided to explain your answers to the questions above. You may also include additional comments for the author, including concerns about dual publication, research ethics, or publication ethics. (Please upload your review as an attachment if it exceeds 20,000 characters)

Reviewer #1: Dear Editor,
thank you for asking me to review this paper submission. Comments:

1) Abstract: Methods not clearly described. How practices were in the Research Network (72 practices were invited to participate but were there lots more practices who were not included? If so, how were the 72 selected?). If 68 practices selected, each contributing 100 patients, why isnt the sample 6800 patients? Were findings 'adjusted' or 'unadjusted', e.g. for age, gender, deprivation; it would be helpful to know in the Abstract if this is a univariate or multivariate analysis.

Response of the authors: We have clarified the practice research network query in the abstract and in the main manuscript as follows:

Abstract

“This was a retrospective study of general practices in a university-affiliated education and research network, consisting of 72 practices.”

Main manuscript

“This study was a retrospective analysis of consultations that took place over a two-year period in general practices associated with the University of Limerick Education and Research Network for General Practice (ULEARN-GP) (25). All 72 practices in the network with a student on placement in 2015/16 were invited to participate.”

We have clarified that 100 patients were selected at random from each practice, resulting in a sample of 6800 patients. A few practices did not provide basic demographic data (age, GMS status) on all 100 patients and these patients were excluded (n =197, 3% of sample). With the word count restriction in the Abstract we have clarified this point in the methods section.

Bivariate associations are given in Table 2 and referral rates are provided by each demographic variable separately (age, gender, etc.). We have clarified this in the Abstract and Methods.

2) Introduction: Reference 5 is cited as evidence of 'the recent funding strategy' for primary care. However, it's worth clarifying that much of this funding in England will be allocated to Primary Care Networks (practices working collaboratively within a locality with a population health agenda) rather than being allocated to individual practices in the traditional way.

Response of the authors: the authors thank the reviewer for pointing out this important distinction. We have clarified in the text as follows:

“In England, a recent funding strategy will support Primary Care Networks, whereby practices work collaboratively within a locality with a population health agenda, and it remains to be seen if this can successfully alleviate the pressure on this key component of the health service.”

3) Introduction: Reference 14 is misquoted. The authors state: 'Multi-morbidity, defined as having at least two chronic diseases (13), is present in over one quarter of adults attending general practice...(14)'. In fact, multimorbidity is present in over a quarter of those REGISTERED at general practices, but account for a much higher proportion of those ATTENDING the practice.

Response of the authors: the authors again thank the reviewer for pointing out this important distinction. We have clarified in the text as follows:

“Multi-morbidity, defined as having at least two chronic diseases (13), is present in over one quarter of adults registered at general practices (and is present in a much higher proportion of those attending general practice) and is associated with higher attendance at general practice and hospital (14).”

4) Introduction: the authors state: 'Therefore, the manual searching of patient records to investigate practice network or national databases is considered to be preferable as they overcome the limitations of coding...'. This provides a strong justification for this study. Studies of coded data are likely to miss cases of multimorbidity (MM). However, the converse should be acknowledged - that a study purely of primary care data will miss emergency presentations to secondary care, which bypass primary care (although these should be recorded retrospectively in primary care case-notes).

Response of the authors: we have acknowledged this point in the introduction:

“Conversely, this method may not account for attendances to emergency departments that did not involve a GP referral, but many of these visits may be recorded in the notes when the relevant hospital discharge letter is filed.”

5) Methods: as per the Abstract, it's not clear if the Network of GP practices consisted of more than 72 practices. Nor why <100 cases were recruited in each practice

Response of the authors: we have clarified that all 72 affiliated practices with a student on clinical placement in 2015/16 were invited. We only included cases that had complete demographic data. We have clarified this point in the methods section and again in the results section.

Methods: “Only data that had complete demographic details were included in the analysis.”

Results: “197 records (3%) were excluded as insufficient demographic data was recorded.”

6) Methods: the authors used Cramer’s V to measure strength of association. This is not a commonly used test. How does it differ from Pearson's r value? My reading is that this study has used univariate parametric correlation coefficients, but I may be mistaken. It is important that the readers know how the data was analysed (univariate presumably, parametric or non-parametric associations?)

Cramer’s V is analagous to Pearson’s r but used for categorical variables (all variables in Table 1) instead of the continuous variables needed for Pearson’s r. Cramer’s V is a measure of the strength of the association between two categorical variables, independent of sample size, and values range from 0 to 1. We have now provided a reference for it (Cohen, J. 1988. *Statistical Power Analysis for the Behavioral Sciences*, 2nd Edition. Routledge).

7) Results: we need a clear definition of MM. Which MM's were included and which were not? Without that list, it's not possible to make sense of the finding: 'The prevalence of multi-morbidity was 38%...'

Response of the authors: we defined multi-morbidity in the introduction as “having at least two chronic illnesses.” [Reference: Le Reste JY, Nabbe P, Manceau B, Lygidakis C, Doerr C, Lingner H, et al.]

Also added to the Methods section: “A list of chronic illnesses, which were defined as a long-term medical condition that cannot be completely cured by medicines, was provided for the students (see supplementary table 1)”. This list is provided now as a supplementary file.

8) Results, Figure 1. The authors state: 'Fig 1 illustrates that with each 10-year increase in age, the rates of attendance to the GP and practice nurse increase.' But they haven't demonstrated this, at least not without further Stats analysis. What if the mean attendance rate confidence intervals overlapped for each 10-year attendance rate value? Without CI's or a test of difference, the authors cannot conclude that there is a difference in these rates. The same applies to the data shown in Figures 2&3. This is an essential revision required before publication.

Response of the authors: We thank the reviewer for this important point. We have now added confidence intervals to all the estimates in Table 2. The confidence intervals are narrow and not overlapping and support the conclusions above.

9) Discussion: The authors do have a key finding that is of importance: 'However, the ratio of attendance to the GP and referral to hospital remained relatively stable between 6.9 and 9.6. These figures indicate that much of the management
of older patients, patients with considerable multimorbidity or polypharmacy are managed in general practice.' This is important and needs emphasis.

Response of the authors: The following has been added under the Conclusions subheading: The implications of this finding are extremely important, as it demonstrates that general practice is bearing most of the burden of increased morbidity and complexity, thereby absorbing excess workload and saving hospital outpatients appointments, emergency department presentations and hospital admissions. Consequently, there are benefits for healthcare economics as well as for the lives of patients who can be managed in the community.

10) Discussion, Strengths and Limitations: the authors state: ' This approach of using multiple sources has been considered advantageous rather than relying on adherence to coding by the GPs'. I suspect the authors don't have the all-important data which would provide a strong justification for their approach. Namely, how much of the data they found were buried away in un-coded data? That would justify manual data searching as in this study, and as opposed to electronic data searching of coded (no freetext) anonymised databases such as CPRD.

Response of the authors: the reviewer is correct on both counts; the proportion of the data found through file-searching of un-coded data would be very helpful and, unfortunately, we did not include the location of recorded information in our protocol.

11) Discussion, Strengths and Limitations: there are many limitations which are not mentioned. A key limitation is how MM was classified (what was and what was not an MM). Why weren't Health Care Assistant consultations included or other healthcare providers within primary care (such as dietitians, midwives, physios etc, if present)? Also, as stated above, what about patients accessing hospital care by bypassing their GP, e.g. emergency admissions - was this data retrospectively captured through inclusion of hospital discharge summaries (this isnt clear in the Methods).
only'

Response of the authors: the authors have added the following sentences to the limitations section to address the points above:

“…the approach may miss self-presentations to the hospital, especially to emergency departments, and such presentations were not included in this study.”

“Irish general practice software systems are not generally used by other healthcare professionals working in primary care such as physiotherapists, dieticians and healthcare assistants; this study involves GPs and practice nurses only. Finally, this study used a definition of multi-morbidity of two or more chronic conditions, based on a pre-defined list that was provided for the students and set by the clinicians involved in the research team, and conditions not on the list were not included in the dataset.”

Reviewer #2: The study objectively shows what many GPs feel on a daily routine: the workload is increasing, the patient cases are becoming more and more complex. Thus, GPs need new tools to be able to treat patients as efficiently and professionally as possible. In my view, the most important result of their study is the constant rate of referrals to hospital. The protective effect for the clinics should not be underestimated. The results of the study are comprehensible and answer the research question. Essentially, I still miss a more detailed description of the data collected:

Response of the authors: we thank the reviewer for positive feedback and for acknowledging the importance of the results in the context of the role of general practice in healthcare systems. Some of the detail is reported in a previous publication emanating from this dataset (Dinsdale, 2021). We will address the specific feedback below.

What were the characteristics of the participating GP practices? Number of doctors, age, number of patients, etc.

Response of the authors: A new table and the following paragraph have been inserted.

All of the practices were mixed public-private, were computerised and had a practice nurse. Table 1 compares the profile of participating practices to the national profile in 2015 (26). Most of the study practices (93%) had a co-operative system of out-of-hours cover, similar to the national profile (92%). Participating practices had higher percentages involved in postgraduate GP training (43% v 22%) and rural location (37% v 21%). In terms of practice size, 68% had between 500 and 1999 patients; 16% were single-handed practices, 31% had two GPs, 24% had three GPs and 29% had four or more GPs.

Is there information on the most common diseases or medication groups that led to the definition of multimorbidity and polypharmacy?

Only a count of the number of chronic conditions and number of medications was recorded.

In which specialist departments were the patients referred?

Response of the authors: The specialties most frequently referred to were internal medicine (29.7% of all referrals), emergency department (11.8%), general surgery (10.1%), orthopaedic surgery (8.0%) and medical assessment unit (6.8%). This has been previously reported in Dinsdale E, Hannigan A, O’Connor R, O’Doherty J, Glynn L, Casey M, Hayes P, Kelly D, Cullen W, O’Regan A. Communication between primary and secondary care: deficits and danger. Family practice. 2020 Feb;37(1):63-8.

Are there diseases that led to particularly frequent referrals - where did outpatient care in GP practices reach its medical limits?

Response of the authors: our data cannot tell us which chronic illnesses led to the most referrals. We have reported the frequency of referral to each hospital department previously.

(Dinsdale E, Hannigan A, O’Connor R, O’Doherty J, Glynn L, Casey M, Hayes P, Kelly D, Cullen W, O’Regan A. Communication between primary and secondary care: deficits and danger. Family practice. 2020 Feb;37(1):63-8.)

Perhaps additional tables could be used to describe the cohort studied even better and thus make it more comparable for other international data!?.

Response of the authors: A new table and the following paragraph have been inserted.

All of the participating practices were mixed public-private, were computerised and had a practice nurse. Table 1 compares the profile of participating practices to the national profile in 2015. Most of the study practices (93%) had a co-operative system of out-of-hours cover, similar to the national profile (92%). Participating practices had higher percentages involved in postgraduate GP training (43% v 22%) and rural location (37% v 21%). In terms of practice size, 75% had between 500 and 1999 patients; 16% were single-handed practices, 31% had two GPs, 24% had three GPs and 29% had four or more GPs.

Note to the reviewers – this study was a retrospective analysis of practice patients and did not distinguish between consultations conducted by various GPs at each practice. We have not, therefore, included gender and age of the GP-tutors as it was not necessarily their own particular consultations (and referrals) that were included.

6. PLOS authors have the option to publish the peer review history of their article ([what does this mean?](https://journals.plos.org/plosone/s/editorial-and-peer-review-process#loc-peer-review-history)). If published, this will include your full peer review and any attached files.


If you choose “no”, your identity will remain anonymous but your review may still be made public.


**Do you want your identity to be public for this peer review?** For information about this choice, including consent withdrawal, please see our [Privacy Policy](https://www.plos.org/privacy-policy).

Reviewer #1: **Yes:** Mark Ashworth

Reviewer #2: **Yes:** Markus Bleckwenn
