# Supplementary material for: How do multi-morbidity and polypharmacy affect general practice attendance and referral rates? A retrospective analysis of consultations
Source: PLoS One. 2022 Feb 3;17(2):e0263258. doi: 10.1371/journal.pone.0263258 (PMC8812985; doi:10.1371/journal.pone.0263258)
Supplement: Attachment — Submitted filename: Response to Reviewers.docx [file pone.0263258.s003.docx]

12/01/2021

Dear editor and reviewers,

The authors are very grateful for the thorough reading and subsequent comments received throughout the review process. The edits from this round of feedback have been helpful, and the paper has been strengthened by them. Please find our point-by-point responses below.

Best wishes,

Dr Andrew O’Regan,

General Practitioner and Senior Lecturer in General Practice

University of Limerick School of Medicine

Review Comments to the Author

Please use the space provided to explain your answers to the questions above. You may also include additional comments for the author, including concerns about dual publication, research ethics, or publication ethics. (Please upload your review as an attachment if it exceeds 20,000 characters)

Reviewer #1: Thank you for asking me to review the revised version of this paper. The revisions have been substantial and greatly improved the standard of this piece of research. I have minor comments only:

Response of the authors: thank you for this acknowledgement and the comments that follow, all of which are helpful.

1) Abstract: this paper is in large part about hospital referral rates for patients with multimorbidity. More precision is required in the Results section of the Abstract which simply says: 'Half of patients had been referred to hospital in the previous two years'. The exact % figure should be given to one decimal place.

Response of the authors: the results section of the abstract now reads: “50.1% of patients had been referred to hospital in the previous two years.”

2) Abstract: a key strength of this work is the use of manual record searching rather than searching of an electronic database. This feature should be added to the Abstract e.g. 'manual record searching'.

Response of the authors: the following clause has been added to the methods section of the abstract: “Through manual record searching…”

3) pg22: minor typo. The sentence says 'aged eligibility' and should read 'age eligibility'. Also, pg 28: 'Analysis of a large clinical practice research database in England reported higher health care utilisation in general practice, higher prescription medications...', should read '...higher number of prescription medications', or similar.

Response of the authors: the typos on page 22 and again on page 28 have been corrected. Thank you for spotting.

4) A further Limitation to be added is that the authors have not identified which LTCs or medications are associated with increased GP/Practice Nurse/Home Visit attendance rates. So we dont know in clinical terms what the drivers are for increased attendance rates (is it mental health conditions, or diabetes, or hypertension, etc etc, all commonly managed in primary care)?

Response of the authors: We agree that this is an important limitation and have added the following sentence to the limitations section: “Finally, during data collection the number of chronic conditions and medications were recorded for each case but not the name of the chronic condition or medication, and, consequently, the analysis cannot identify which chronic conditions or medications were associated with attendance.”
